# Single and Repeated Doses of EGb 761^®^ do not Affect Pharmacokinetics or Pharmacodynamics of Rivaroxaban in Healthy Subjects

**DOI:** 10.3389/fphar.2022.868843

**Published:** 2022-04-20

**Authors:** Robert Hoerr, Andrea Zimmermann, Friedeborg Seitz, Angelika Dienel

**Affiliations:** ^1^ Dr. Willmar Schwabe GmbH & Co. KG, Research and Development, Karlsruhe, Germany; ^2^ Dr. Willmar Schwabe GmbH & Co. KG, Biometry Department, Karlsruhe, Germany; ^3^ CRS Clinical Research Services Mannheim GmbH, Mannheim, Germany; ^4^ Dr. Willmar Schwabe GmbH & Co. KG, Clinical Research, Karlsruhe, Germany

**Keywords:** *Ginkgo biloba* extract, rivaroxaban, drug-drug interaction, EGb 761®, pharmacokinetics, pharmacodynamics, healthy subjects

## Abstract

The present drug-drug interaction study investigates whether single or repeated doses of 240 mg *Ginkgo biloba* extract EGb 761^®^ alter the pharmacokinetics or pharmacodynamics of rivaroxaban in healthy subjects. This was a single-centre, two-period, fixed-sequence trial. In Period 1, rivaroxaban was taken alone. In Period 2, rivaroxaban was given on the first and last of 8 days of EGb 761^®^ treatment. Plasma concentrations of rivaroxaban and anti-Factor Xa activity were determined until 48 h after each rivaroxaban intake. The data of forty-one healthy subjects (25 males, 16 females) aged 21–70 years were evaluable. Geometric mean ratios (90% confidence intervals) for rivaroxaban administered concomitantly with a single or multiple doses of EGb 761^®^ vs. rivaroxaban administered alone were 97.97 (91.78, 104.58) and 96.78 (90.67, 103.31) for maximum concentration (C_max_), 98.55 (94.43, 102.84) and 97.82 (93.73, 102.08) for area under the concentration-time curve (AUC_0-∞_) of rivaroxaban in plasma (primary endpoints), 98.19 (92.00, 104.80) and 99.78 (93.43, 106.55) for maximum effect (E_max_), 99.46 (93.63, 105.66) and 99.12 (93.25, 105.35) for area under the effect curve (AUEC_0-48_). All 90% confidence intervals were within the prespecified range of 80%–125%. Neither adverse events related to haemorrhages nor clinically significant findings in haematology or coagulation parameters were observed. The treatments were safe and well-tolerated. Single and repeated doses of EGb 761® neither affect plasma concentrations of rivaroxaban nor anti-Factor Xa activity in healthy subjects.

## Introduction

EGb 761^®^ ^1^ is a dry extract from Ginkgo biloba leaves [drug-extract ratio 35–67:1, extraction solvent: acetone 60% (w/w)] adjusted to 22–27% ginkgo flavonoids calculated as ginkgo flavone glycosides and 5.4–6.6% terpene lactones consisting of 2.8–3.4% ginkgolides A, B, C and 2.6–3.2% bilobalide and containing less than 5 ppm ginkgolic acids. The main pharmacological effects of EGb 761^®^ are the protection against peroxidation of brain lipids and mitochondrial DNA, improvement in mitochondrial function, stimulation of neurogenesis and synaptogenesis, and improvement of microcirculation (Nowak et al., 2021; Tomino et al., 2021). The extract affects several neurotransmitter pathways involved in cognitive functioning (Nowak et al., 2021). Medicinal products containing EGb 761^®^ as active substance are widely used to treat cognitive impairment and dementia (Gauthier and Schlaefke, 2014; Liu et al., 2020).

Herbal preparations from Ginkgo biloba leaves in general are suspected to cause interactions, namely *via* cytochrome P-450 (CYP-450) enzymes and transporters (e.g., P-glycoprotein, P-gp). Case reports on bleeding events associated with Ginkgo preparations in general are discussed in the scientific literature as hints for pharmacodynamic interactions with antiplatelet drugs (e.g., by inhibition of platelet-activating factor) or anticoagulants (Ernst et al., 2005; Hoban et al., 2019). Plant extracts are composed of numerous different molecules and the manufacturing process, which includes extraction and purification, determines their final composition, efficacy, safety, and interaction potential (Gawron-Gzella et al., 2010; Wohlmuth et al., 2014). For EGb 761^®^, the potential for clinically relevant drug-drug interactions (DDIs) is reported to be to be negligible (Hermann and von Richter, 2012; Unger, 2013). In well-controlled trials, no relevant interactions of EGb 761^®^ with platelet anti-aggregatory effects of acetylsalicylic acid were identified (Wolf, 2006; Gardner et al., 2007). A dedicated interaction study did not provide evidence of relevant DDIs between EGb 761^®^ and warfarin (Jiang et al., 2005). Furthermore, EGb 761^®^ did not interact pharmacokinetically or pharmacodynamically with ticlopidine (Kim et al., 2010). A study conducted in India found that an unidentified extract amplified the bleeding time-prolonging effect of cilostazol, whereas the extract itself neither affected platelet aggregation nor enhanced antiplatelet effects of cilostazol Aruna and Naidu, 2007 (study conducted in India). In another study, concomitant intake of a product marketed in Korea did not lead to pharmacokinetic interactions with cilostazol, but caused a slight numeric, non-significant difference of adenosine diphosphate**-**induced aggregation while bleeding time was not enhanced Kim et al., 2014, study using a Korean product. However, no studies were conducted on possible interactions of EGb 761^®^ with direct anticoagulants such as rivaroxaban so far.

Rivaroxaban is an oral, direct Factor Xa inhibitor targeting free and clot-bound Factor Xa and Factor Xa in the prothrombinase complex (Mueck et al., 2013a). It is approved for the treatment and prevention of various thromboembolic disorders, e.g., venous thromboembolism, pulmonary embolism, or stroke. Orally administered rivaroxaban is readily absorbed. Under fed conditions, the exposure levels were proportional to the dose administered in the range of 10–20 mg. The pharmacodynamic effect of rivaroxaban is closely correlated with its plasma concentration. About 36% of the rivaroxaban dose is eliminated as unchanged drug in the urine. Interaction studies suggest that P-glycoprotein (P-gp) and breast cancer resistance protein are involved as transporters in active renal secretion. Approximately two-thirds of a dose is subject to metabolic degradation. CYP3A4 accounts for approximately 18%, CYP2J2 for approximately 14%, and non-CYP mediated hydrolysis of the amide bonds for about 14% of the rivaroxaban elimination. The resulting metabolites are excreted via renal and hepatobiliary routes (Mueck et al., 2013a; Harder, 2014).

The present DDI study was conducted to examine whether EGb 761^®^ alters the pharmacokinetics (PK) or pharmacodynamics (PD) of rivaroxaban.

## Materials and Methods

The study (EudraCT No. 2019-004672-19) was conducted in Germany in the years 2020–2021 in order to examine PK, PD, as well as safety and tolerability of rivaroxaban administered concomitantly with single and multiple doses of EGb 761^®^. The protocol, subject information, and informed consent form were reviewed and approved by an independent ethics committee. The study was planned, conducted, analysed, and reported in accordance with the principles of Good Clinical Practice, the Declaration of Helsinki, and national laws on clinical trials. To participate, subjects had to provide informed consent before the start of any trial-related procedure.

### Participants

Healthy Caucasian males or females of at least 18 years of age, with a body mass index (BMI) of 18.0–29.9 kg m^−2^ could be enrolled. Their eligibility was evaluated by extensive screening within 2–7 days prior to the first profiling day (Day 1). Screening consisted of medical history recording, review of co-medication, physical examination, vital functions, 12-lead electrocardiogram, laboratory tests (haematology, coagulation, clinical chemistry, C-reactive protein, urinalysis, hepatitis and human immunodeficiency virus serology, SARS-CoV-2 viral testing, tests for occult faecal blood), breath test for alcohol, urine dipstick test for drugs, and pregnancy test (if applicable).

### Study Design and Procedures

**FIGURE 1:**
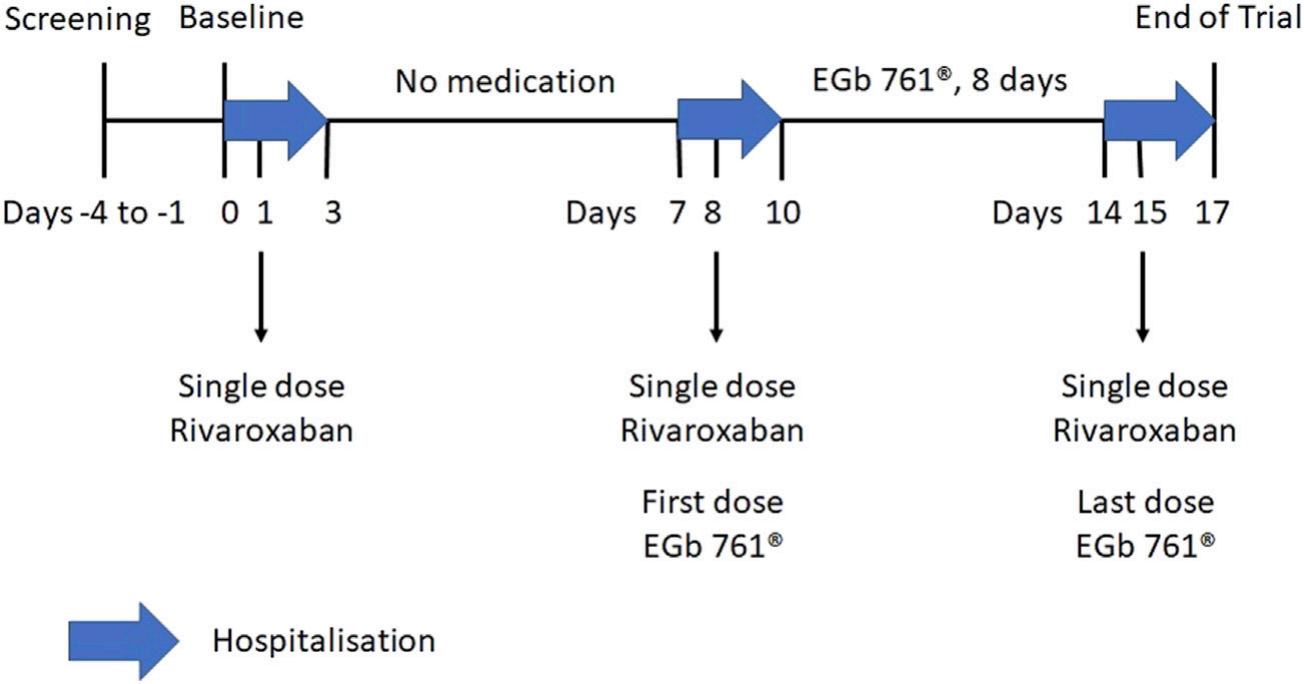
Overview of the treatment periods in the drug-drug-interaction trial.

The single-centre, open-label, single-arm trial comprised two treatment periods. On Day 1 of Period 1, subjects received a single dose of 20 mg rivaroxaban (Xarelto^®^; Bayer AG, Leverkusen, Germany). In Period 2, 240 mg EGb 761^®^ (Tebonin^®^ konzent; Dr. Willmar Schwabe GmbH and Co. KG, Karlsruhe, Germany) was taken once daily for 8 days (Day 8 to Day 15). Rivaroxaban was given on Day 8 and Day 15. The 8 day period for EGb 761^®^ intake was chosen in order to be reasonably sure that the numerous components of the extract were in a steady state and that any inducing effect had time to unfold. Plasma concentrations of rivaroxaban and anti-Factor Xa (anti-FXa) activity were determined until 48 h after each rivaroxaban administration on Day 1, Day 8, and Day 15 (Figure 1). For the pharmacokinetic and pharmacodynamic profiling of rivaroxaban, the subjects were under close medical surveillance as they stayed in a specialised clinical research unit from the evening before exposure until 48 h after.

Each rivaroxaban dose was taken orally with 240 ml water after an overnight fast, at the end of a light breakfast. The mouth and hands of the participants were checked after dosing. Except for water after 1 h, no food was allowed until 4 h post-dose.

EGb 761^®^ had to be taken once daily with 240 ml water in the morning after an overnight fast. From Day 8 to Day 10 and on Day 15, the tablets were taken at the trial site and from Day 11 to Day 14 at home. On Day 8 and Day 15, EGb 761^®^ was taken 1 hour before the intake of rivaroxaban.

During in-house stays, subjects were only allowed food and beverages provided by the trial site. Foods and drinks containing grapefruit, Seville oranges, tangelos, or pomelos were not allowed on medication days. The intake of other medicinal drugs as well as smoking were not allowed throughout the study. Coffee, xanthine-containing drinks, or alcohol were not allowed throughout the in-house stays, but moderate amounts were permitted during the ambulatory phase. In order to avoid positive drug test results, the consumption of poppyseed-containing foods or beverages was prohibited within 48 h before admission until discharge from the trial site.

On Day 1, Day 8, and Day 15, venous blood was taken before and 0.5, 1, 1.5, 2, 3, 4, 6, 8, 12, 24, and 48 h after intake. Blood was collected into K_2_-EDTA S-Monovettes and placed in an ice bath immediately. Within 30 min, the samples were centrifuged at 3,000 rpm for 10 min at 4°C. Plasma was then harvested and stored in cryotubes at −20 ± 5°C until the assay was carried out.

Plasma rivaroxaban concentrations were determined in 100 μL plasma specimens. The drug was extracted by protein precipitation with subsequent high-pressure liquid chromatography (HPLC) coupled to tandem mass spectrometry (MS/MS) (Rohde, 2008). The method for measurement of rivaroxaban in plasma samples was validated following regulatory requirements of the European Medicines Agency (EMA, 2011). The standard curve was linear in the range of 2–500 ng/ml. The inter-assay bias ranged from −8.4% to −10.8% for the highest and lowest calibrator, respectively. The inter-assay imprecision ranged from 3.0% (highest concentrate of the analyte) to 4.2% (medium concentrate of the analyte). Incurred sample re-analysis showed that 92% of the samples fulfilled acceptance criteria.

For each dosing of rivaroxaban, the plasma concentrations were determined non-compartmentally as maximum plasma concentration (C_max_), time of C_max_ after dosing (t_max_), area under the concentration-time curve in plasma (AUC), i.e., from zero up to the last quantifiable concentration (AUC_0–tz_), AUC extrapolated to infinity (AUC_0–∞_), and apparent terminal half-life (t_1/2λz_). C_max_ and AUC_0-∞_ of rivaroxaban alone or together with single and multiple doses of EGb 761^®^ were the primary PK outcome variables.

PD of rivaroxaban were characterised by the concentration equivalents of the anti-FXa activity using a commercial one stage test method (HemosIL by Instrumentation Laboratory Company - Bedford, MA 01730-2443, United States): Residual FXa in patient plasma reacted with a synthetic chromogenic substrate which was measured photometrically at 405 nm and was inversely proportional to the rivaroxaban level in the sample. The measurements were carried out automatically after the assay was calibrated with specific rivaroxaban calibrators (HemosIL by Instrumentation Laboratory Company). In the following, anti-FXa activities are therefore reported as rivaroxaban concentrations (ng/ml), with which they relate linearly in the therapeutic range (Douxfils et al., 2013; Beyer et al., 2016; Ikeda and Tachibana, 2016). The lower limit of quantification of the method was 20 ng/ml.

Pharmacodynamic objective was to investigate the PD of rivaroxaban when given together with single and multiple doses of EGb 761^®^, respectively, as compared to rivaroxaban given alone as single oral dose. Maximum measured effect (E_max_) and area under the effect-time curve from 0 to 48 h after administration (AUEC_0-48_) of anti-FXa activity after each rivaroxaban administration were chosen as endpoints in alignment with published studies (Kreutz et al., 2017; Kubitza et al., 2018).

### Statistical Analysis

The sample size calculation was based on the PK parameters C_max_ and AUC and the intra-subject coefficient of variation (CV) was estimated from published data of interaction studies with rivaroxaban (Mueck et al., 2013b). For an intra-subject CV of 30% or less and geometric mean ratios deviating by not more than 5%, a sample size of 42 subjects is required to conclude “no relevant effect” with a power of at least 80% and accounting for possible premature discontinuations. As no specific boundaries were known, the classical default no-effect boundaries of 80–125% were applied (Food and Drug Administration, 2020).

The statistical model used for the analysis of the primary PK and PD endpoints was an analysis of variance model on the logarithmic scale. The main PK and PD characteristics of rivaroxaban along with the first and last dose of concomitant repeated dosing of EGb 761^®^ (Day 8 and 15, respectively) were compared to the administration of rivaroxaban alone (Day 1). Therefore, the analysis of variance-derived point and 90% confidence interval (CI) estimates of the treatment ratios (Day 8/Day 1 and Day 15/Day 1) were back-transformed from the corresponding mean differences of the log-normal-transformed data.

PK or PD interaction between rivaroxaban and single or repeated dosing of EGb 761^®^ (Day 8 and Day 15, respectively) was considered absent if the boundaries of the 90% CI for the geometric mean ratios of the primary PK and PD endpoints (C_max_, AUC_0-∞_, E_max_ and AUEC_0-48_) were within the widely accepted no-effect boundaries (80–125%).

All statistical calculations were carried out using SAS 9.4 (SAS Institute Inc., Cary, NC, United States). The PK and PD parameters were calculated using WinNonlin 8.1 (Phoenix^®^ WinNonlin^®^, Certara United States, Inc., Princeton, NJ, United States).

## Results

### Subjects

Forty-two healthy subjects (26 men, 16 women) were included with a mean age of 46.7 ± 14.4 years (range: 21–70 years), a mean body weight of 77.9 ± 13.1 kg (range: 52.3–115.0 kg), and a mean body mass index of 25.0 ± 2.6 kg m^−2^ (range: 20.2–29.9 kg m^−2^). A 54 year-old man discontinued the study prematurely on Day 8 due to elevated blood pressure (details see below). Therefore, 41 subjects were evaluable for PK and PD.

### PK and PD

**FIGURE 2:**
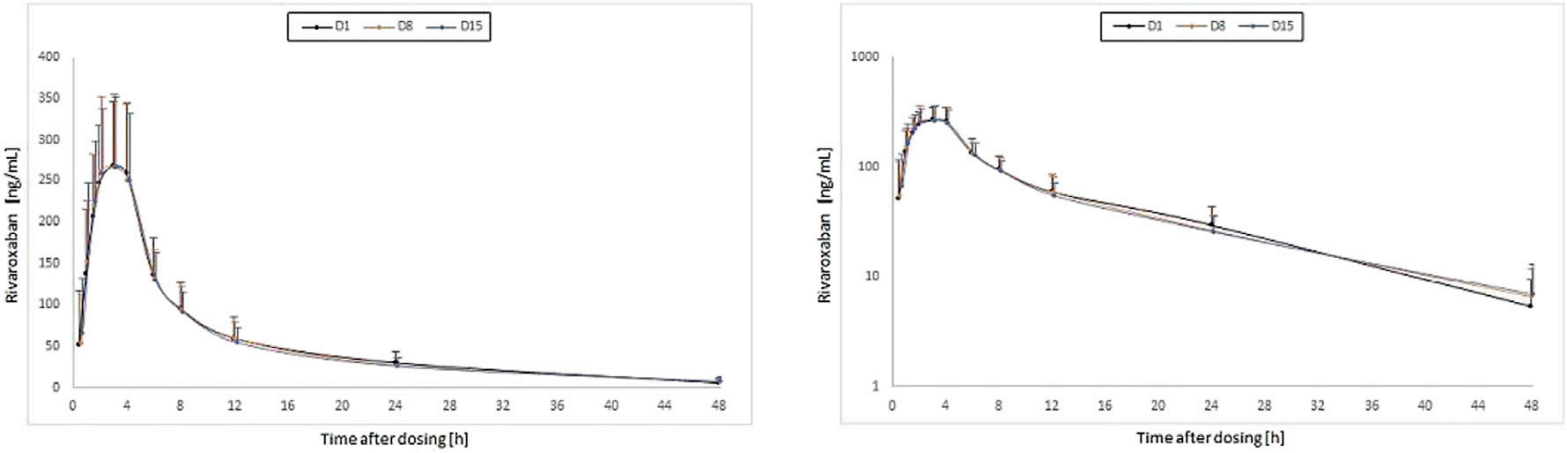
Time courses of the plasma concentrations of rivaroxaban (means, standard deviations; left: linear axis; right: log-linear axis) for single doses of 20 mg rivaroxaban at baseline (Day 1 [D1]) and concomitantly with the first (Day 8 [D8]) and the last dose (Day 15 [D15]) of a 1 week treatment with once-daily dosing of 240 mg EGb 761^®^ in 41 healthy subjects.

There was a close agreement between the PK profiles when rivaroxaban was taken alone (Day 1) or when taken after EGb 761^®^ as single dose (Day 8) or EGb 761^®^ as multiple doses (Day 15) (Table 1, Table 2). C_max_ and AUC_0-∞_ of rivaroxaban in plasma showed no marked differences at Day 1, Day 8, and Day 15 (Table 2, Figure 2).

**TABLE 1:** PK parameters of rivaroxaban (time courses of the plasma concentrations) and PD parameters (anti-FXa activity measured by chromogenic assay).

		Profiling day		
	Unit	Day 1	Day 8	Day 15
PK parameters of rivaroxaban				
		N: 41	N: 41	N: 41
C_max_	[ng/mL]^a^	281.8 (28.3)	276.0 (35.9)	272.7 (32.9)
AUC_0-∞_	[ng[Table-fn Tfn1]h/mL]^a^	2679 (27)	2640 (28)	2620 (27)
AUC_0-tz_	[ng[Table-fn Tfn1]h/mL]^a^	2542 (28)	2472 (28)	2467 (26)
T_max_	[h]^b^	3.00 (1.00–4.02)	2.08 (1.00–4.02)	2.05 (0.50–4.00)
t_½_	[h]^c^	10.78 ± 3.87	11.62 ± 3.80	11.80 ± 3.78
PD parameters of rivaroxaban				
		N: 41	N: 41	N: 40^d^
E_max_	[ng/mL]^a^	202.3 (27.9)	198.6 (35.7)	200.1 (34.2)
AUEC_0-48_	[ng[Table-fn Tfn1]h/mL]^a^	1555 (34)	1547 (33)	1528 (37)

a Geometric mean (geometric coefficient of variation [%]).

b Median (range: minimum—maximum).

c Arithmetic mean ± standard deviation.

d Data missing in one subject due to technical reasons.

**TABLE 2:** Geometric mean ratio (90% confidence interval) of PK and PD parameters after single (Day 8) or repeated intake of 240 mg EGb 761^®^ (Day 15) relative to the intake of rivaroxaban alone (Day 1).

	Ratio Day 8/Day 1 (%)	Ratio Day 15/Day 1 (%)	Multiplicative Coefficient of Variation (%)
PK parameters of rivaroxaban			
C_max_	97.97 (91.78–104.58)	96.78 (90.67–103.31)	17.90
AUC_0-∞_	98.55 (94.43–102.84)	97.82 (93.73–102.08)	11.65
PD parameters of rivaroxaban			
E_max_	98.19 (92.00–104.80)	99.78 (93.43–106.55)	17.86
AUEC_0-48_	99.46 (93.63–105.66)	99.12 (93.25–105.35)	16.56

**FIGURE 3:**
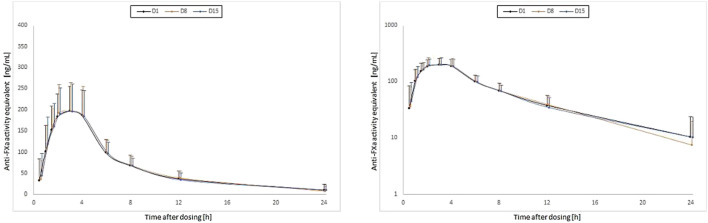
Time courses of the anti-FXa activity (means, standard deviations; left: linear axis; right: log-linear axis) for single doses of 20 mg rivaroxaban at baseline (Day 1 [D1]) and concomitantly with the first (Day 8 [D8]) and the last dose (Day 15 [D15]) of a 1 week treatment with once-daily dosing of 240 mg EGb 761^®^ in 41 healthy subjects.

The descriptive statistics of the PD parameters are shown in Table 1; the comparison of the treatments in Table 2. E_max_ and AUEC_0-48_ showed no marked differences at Day 1, Day 8, and Day 15 (Table 2, Figure 3). The intake of EGb 761^®^ as single dose or repeated doses had no relevant impact on E_max_ or AUEC_0-48_ of rivaroxaban, as the estimated mean ratios Day 8/Day 1 and Day 15/Day 1 were between 96.78 and 99.46.

The time courses of the geometric mean for the anti-FXa activity of rivaroxaban measured as concentration analogues by the chromogenic assay are shown in Figure 3. There was an apparent overlap of the PD profiles of rivaroxaban determined at Day 1, Day 8, and Day 15.

### Safety and Tolerability

Twenty-seven non-serious adverse events (AEs) were reported for 17 of 42 participants. No bleeding events or AEs related to haemorrhages were observed. There were no changes in relevant laboratory parameters (erythrocyte count, haemoglobin, haematocrit, platelet count, urine haemoglobin, or urine erythrocytes) that would suggest blood loss after administration of rivaroxaban in combination with EGb 761^®^.

With 10 events in eight subjects, headache was the predominant event. AEs were mostly mild (23 AEs in 15 subjects). Four AEs in three subjects were of moderate intensity: Fatigue and nausea on Day 8 occurred in one subject. Back pain with onset prior to Day 1 was also present in one subject. Moreover, elevated blood pressure (168/103 mmHg with a pulse rate of 69 bpm vs. 139/97 mmHg and 78 bpm at baseline) in a 54 year-old male in the morning of Day 8, 16 min after intake of the first dose of EGb 761^®^ and before intake of rivaroxaban, led to premature discontinuation. The man recovered rapidly without intervention and the causal relationship between the event and EGb 761^®^ was assessed as unlikely.

Back pain of moderate intensity and mild headache required the intake of 500 mg paracetamol. All AEs had resolved at the end of the trial except for a case of skin irritation that lasted for a few more days after Day 17, when an interventional peeling was applied.

## Discussion

The PK parameters determined in this study were in line with published data (Mueck et al., 2013a; Kreutz et al., 2017). Their analysis showed that co-administration of EGb 761^®^ had no effect on the PK of rivaroxaban. The 90% CIs for the comparisons of AUC_0-∞_ and C_max_ of rivaroxaban in plasma were close to 100% and within the widely accepted no-effect boundaries of 80%–125% (EMA, 2012; Food and Drug Administration, 2020).

The close relationship between plasma concentration of rivaroxaban and anti-FXa activity is reflected in the PD variables E_max_ and AUEC_0-48_. Both PD variables mirrored the results for AUC_0-∞_ and C_max_ of rivaroxaban in plasma, which is in line with published data (Kreutz et al., 2017). The 90% CIs for the PD comparisons for E_max_ and AUEC_0-48_ were also within the prespecified limits. The present study thus demonstrates that single or repeated doses of 240 mg EGb 761^®^ once daily do not affect the PK or PD of single doses of 20 mg rivaroxaban.

The composition and interaction potential of other Ginkgo extracts may differ considerably from that of EGb 761^®^ (Gawron-Gzella et al., 2010), as the composition of a herbal extract strongly depends on the manufacturing process. The lack of impact on the PK of rivaroxaban seen for EGb 761^®^ in the present study is therefore not contradictory to the strong P-gp-inducing effect reported for other Ginkgo extracts (Fan et al., 2009).

*Ginkgo biloba* extracts—not necessarily EGb 761^®^—have been associated with bleeding events due to their pharmacodynamic effects on the one hand and due to DDIs with anticoagulant drugs on the other hand. These reports originate primarily from the spontaneous reporting of suspected adverse drug reactions (Ernst et al., 2005; Hoban et al., 2019). Post-marketing data in general should be interpreted with caution since they are not always well documented and prone to reporting bias. They should therefore only be considered as clinically relevant if they have been confirmed by well-designed, dedicated interaction studies.

In a double-blind, placebo-controlled trial investigating EGb 761^®^ in patients with Alzheimer’s disease, prothrombin time, activated partial thromboplastin time, international normalized ratio, and bleeding time were measured at baseline and after 6 and 26 weeks of treatment (Kloft and Hoerr, 2021). In this trial, no relevant changes were found for coagulation parameters and bleeding time. Moreover, no indication for pharmacodynamic interactions with warfarin or acetylsalicylic acid were found. In another study, intraindividual comparisons of prevalence estimates for bleeding events of patients taking antidementia agents and any antiplatelet or anticoagulant drugs were performed by analysis of more than 300,000 datasets of a German prescription database (Gaus et al., 2005). Analysis results showed that intake of EGb 761^®^ together with any antiplatelet or anticoagulant drugs did not increase the risk of bleeding events. The present study adds to this evidence in demonstrating that EGb 761^®^ does neither interact with the pharmacokinetics nor with the pharmacodynamics (anti-FXa effects) of rivaroxaban. No indication for an additional bleeding risk was found.

## Data Availability

The datasets presented in this article are not readily available because of data protection laws. Requests to access the datasets should be directed to Robert Hoerr, robert.hoerr@schwabe.de.

## Footnotes

1 EGb 761^®^ is a refined and quantified Ginkgo dry extract [Ph. Eur] developed and manufactured by Dr. Willmar Schwabe, Karlsruhe, Germany.
